# Supplementary figures and images for: Expression analysis of microbial rhodopsin-like genes in Guillardia theta
Source: PLoS One. 2020 Dec 3;15(12):e0243387. doi: 10.1371/journal.pone.0243387 (PMC7714340; doi:10.1371/journal.pone.0243387)

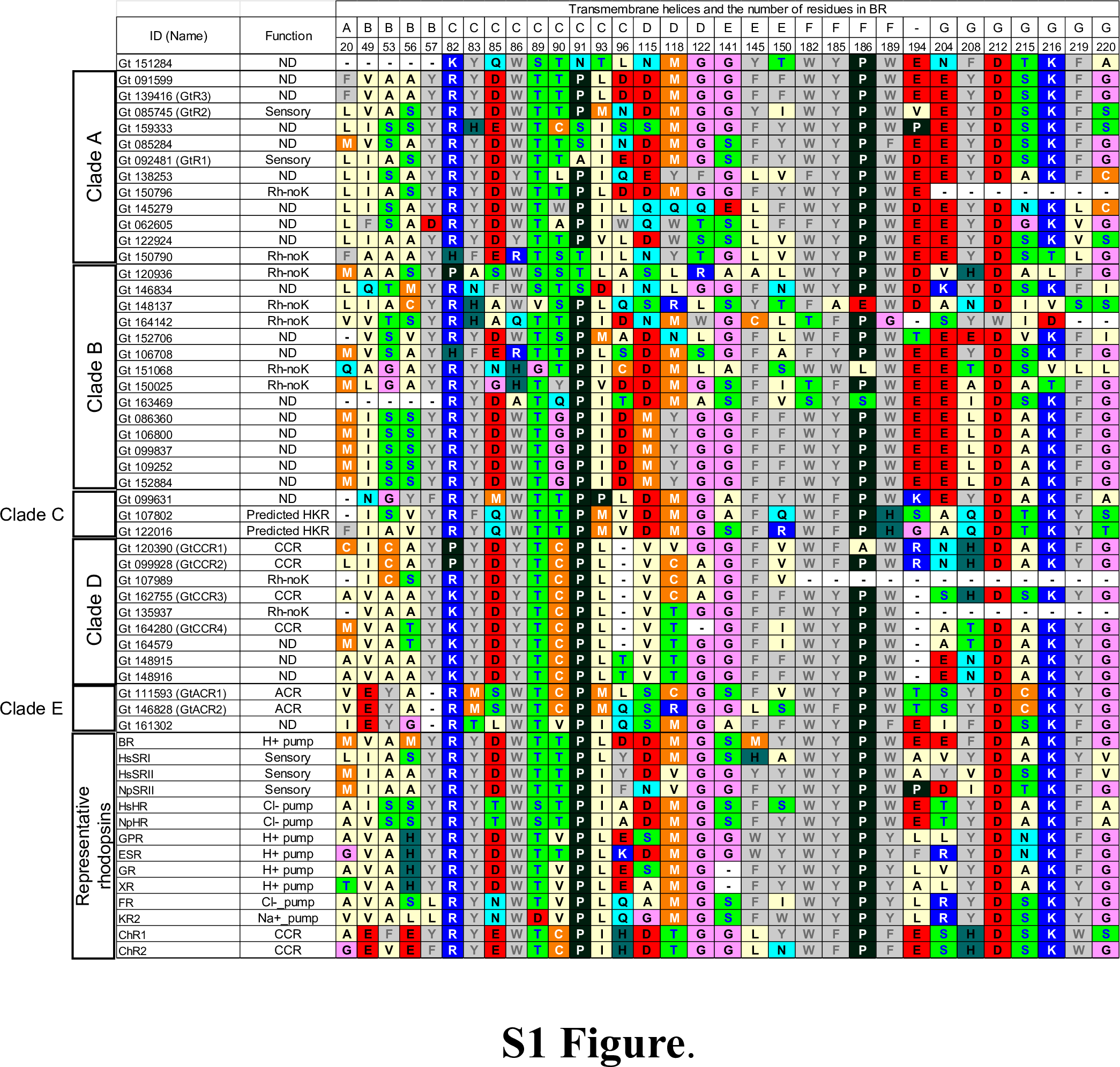

Supplement: S1 Fig — The name of representative rhodopsins indicated in the legend of Fig 1. The color of cells is described below. Negatively charged residues: red, positively charged residues: blue, aromatic residues: grey, polar residues: light green, non-polar residues: pale yellow, methionine and cysteine: orange, histidine: dark green, glutamine and asparagine: light blue, glycine: pink, proline: black. (TIF) [file pone.0243387.s001.tif]

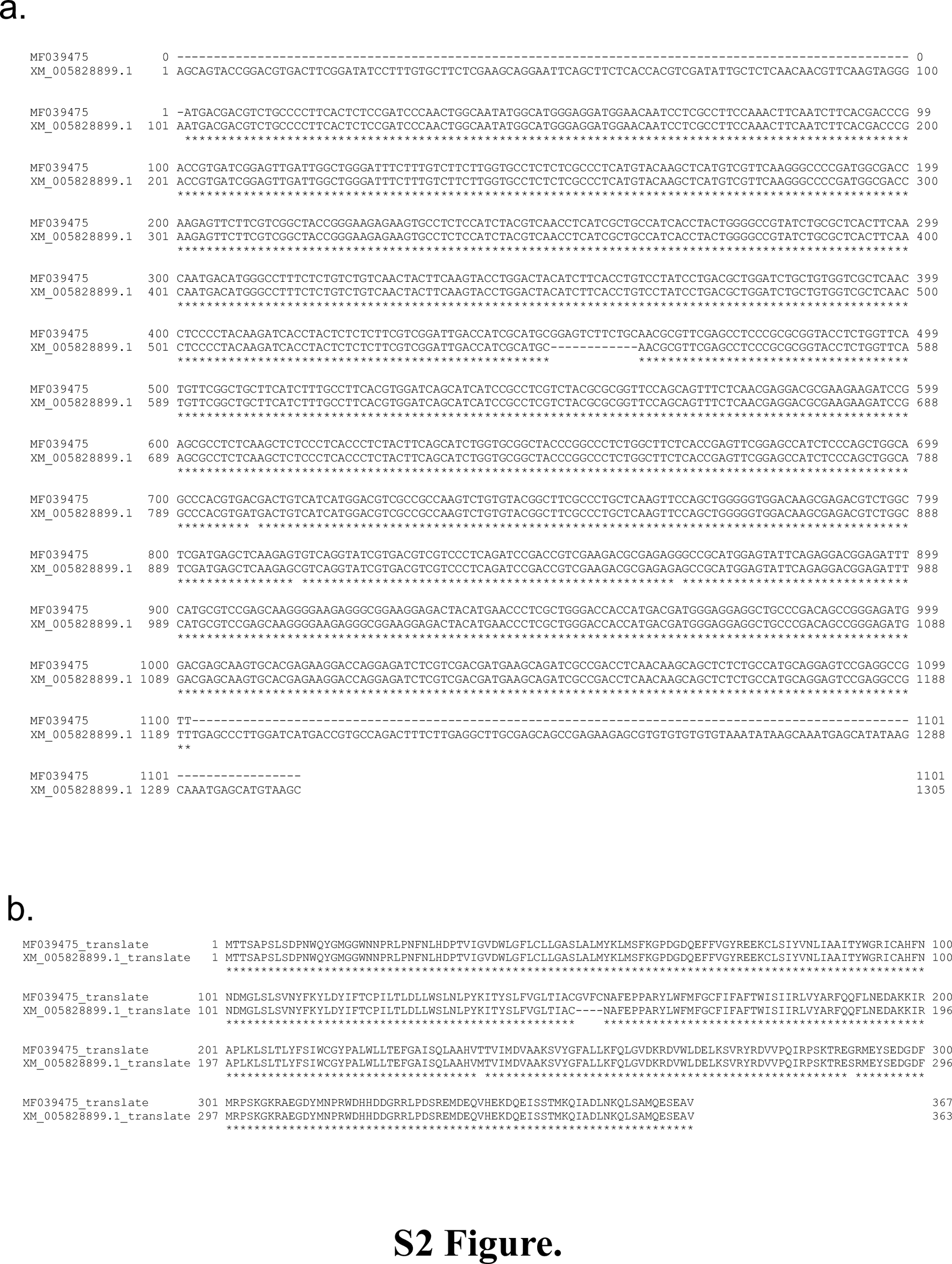

Supplement: S2 Fig — a. Sequence comparison with model transcripts. b. Predicted amino acid sequence derived from re-analyzed sequence compared with that of model transcripts. MF039475: the sequence which we determined, XM_005828899.1: the model transcript of Gt_164280. Asterisks indicate that the two sequences are identical at the corresponding sites. (TIF) [file pone.0243387.s002.tif]

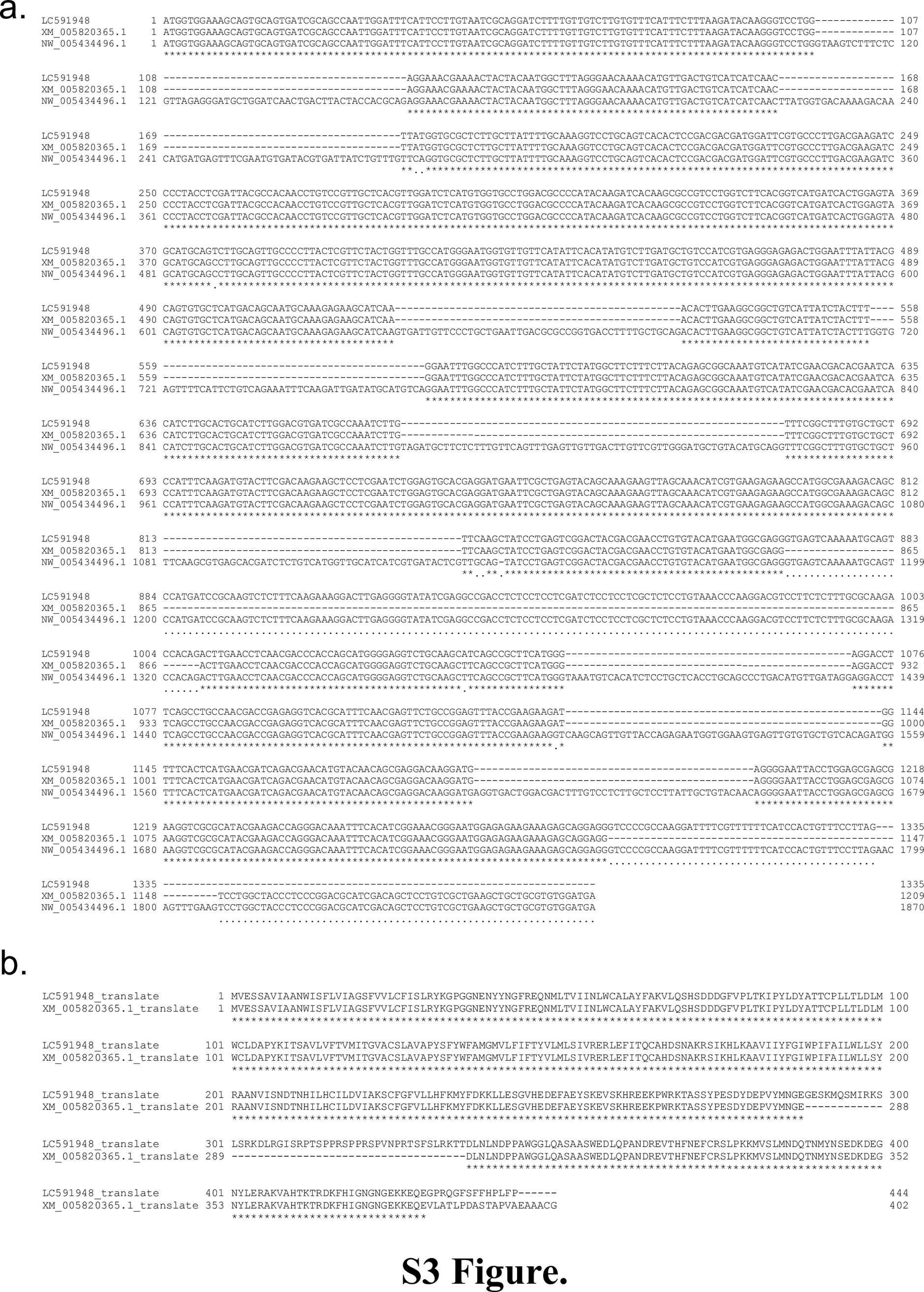

Supplement: S3 Fig — a. Sequence comparison with model transcripts and genomic sequence. b. Predicted amino acid sequence derived from re-analyzed sequence compared with that of model transcripts. LC591948: the sequence which we determined, XM_005820365.1: the model transcript of Gt_120390, NW_005464496.1: the genomic sequence of Gt_120390. Asterisks indicate that the all sequences are identical at the corresponding sites. Dots indicate that the two of three sequences are identical at the corresponding sites. (TIF) [file pone.0243387.s003.tif]

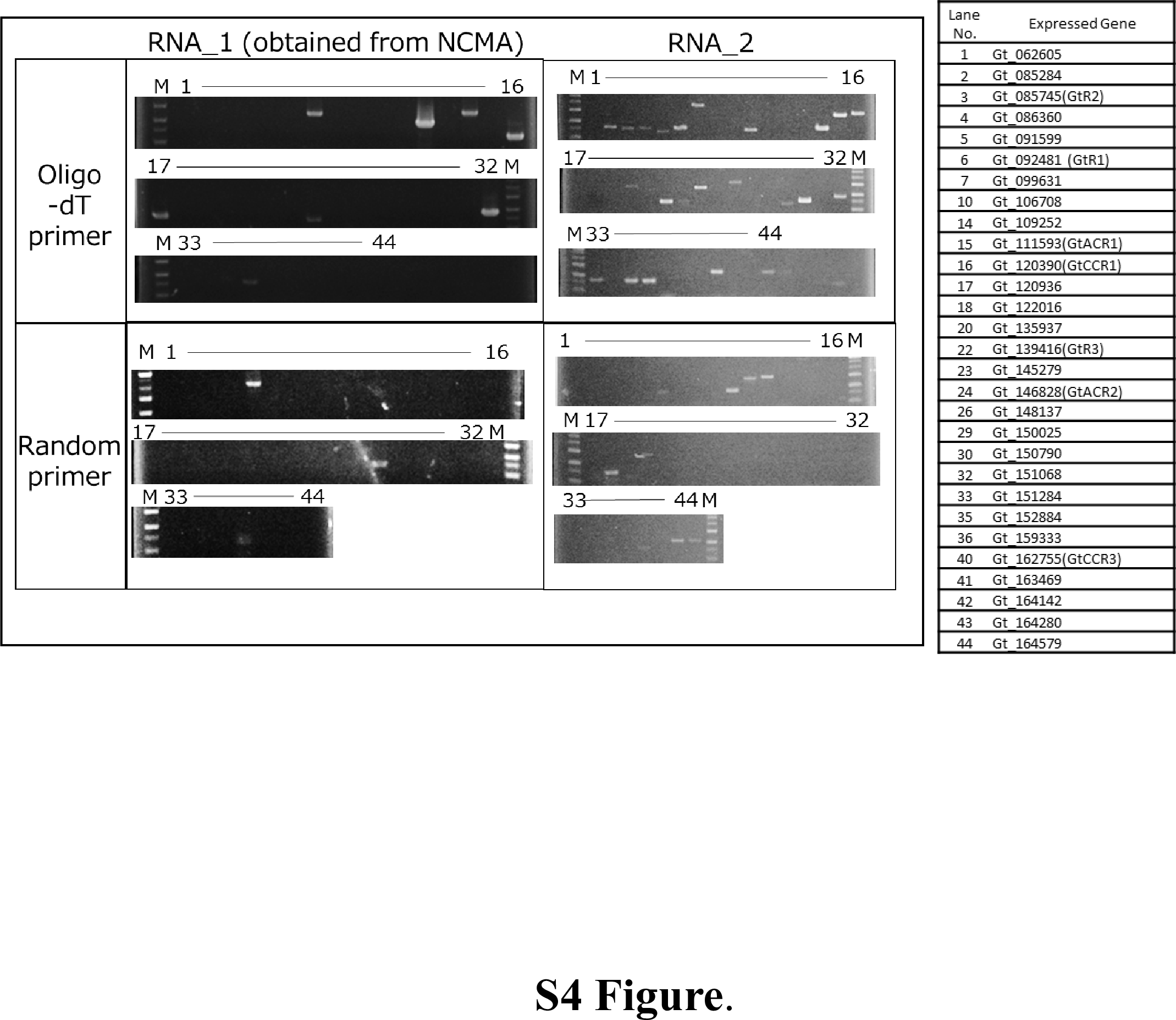

Supplement: S4 Fig — PCR products were detected by electrophoresis on 1% agarose gel. The lane number and ID of the expressed genes are shown in the right table. The letter and numbers are described below. M: DNA marker, 1: Gt_062605, 2: Gt_085284, 3: Gt_085745, 4: Gt_086360, 5: Gt_091599, 6: Gt_092481, 7: Gt_099631, 8: Gt_099837, 9: Gt_099928, 10: Gt_106708, 11: Gt_106800, 12: Gt_107802, 13: Gt_107989, 14: Gt_109252, 15: Gt_111593, 16: Gt_120390, 17: Gt_120936, 18: Gt_122016, 19: Gt_122924, 20: Gt_135937, 21: Gt_138253, 22: Gt_139416, 23: Gt_145279, 24: Gt_146828, 25: Gt_146834, 26: Gt_148137, 27: Gt_148915, 28: Gt_148916, 29: Gt_150025, 30: Gt_150790, 31: Gt_150796, 32: Gt_151068, 33: Gt_151284, 34: Gt_152706, 35: Gt_152884, 36: Gt_159333, 37: Gt_161302, 38: Gt_162755, 39: Gt_163469, 40: Gt_164142, 41: Gt_164280, 42: Gt_164579, 43: Gt_161042, 44: Gt_162503. (TIF) [file pone.0243387.s004.tif]
