# Supplementary material for: Expression analysis of microbial rhodopsin-like genes in Guillardia theta
Source: PLoS One. 2020 Dec 3;15(12):e0243387. doi: 10.1371/journal.pone.0243387 (PMC7714340; doi:10.1371/journal.pone.0243387)
Supplement: Attachment — Submitted filename: Konno_et_al_Response to reviewers.docx [file pone.0243387.s006.docx]

**Response to Reviewer 1:**

Thank you for your crucial reading of our manuscript and nice comments on it. We reply to your comments as follows.

[Comment 1]

This is a timely report on expression of rhodopsin genes in Guillardia theta under nitrogen depletion.

Only two remarks to the authors:

1. G. theta carries some very strange heliorhodopsin-like genes. Please clearly indicate in the manuscript that those were not looked after. Or if you did measure heliorhodopsin transcripts please report it.

[Reply]

As you mentioned, *G. theta* carries two heliorhodopsin-like genes. In this manuscript, we did not focus on these in the expression analysis, so we have indicated the following in “Results”.

Line 151-153: “*Guillardia theta* also carries two heliorhodopsin-like genes (XM_005821825 and XM_005823076); however, we did not analyze the expression of these heliorhodopsins in this study*.*”

[Comment 2]
2. In the tree in figure 1. please clearly mark G. theta rhodopsins for which known activity was previously reported.

[Reply]

As you mentioned, we marked *G. theta* rhodopsin whose activity has already been reported using the red underline in figure 1.

**Response to Reviewer 2:**

Thank you for your crucial reading of our manuscript and nice comments on it. We will reply to your comments as follows.

[Comments]
The manuscript by Konno et al. entitled “Expression analysis of microbial rhodopsin-like genes in Guillardia theta” reports exactly what is stated in its title. G. theta ACRs have been widely used to control neurons with light, but little is known about their function in the alga itself, and even less is known about other rhodopsin genes in this organism. The Authors have determined expression levels of G. theta rhodopsin genes under normal and nitrogen-deficient conditions and found that nitrogen depletion promoted expression of some rhodopsin genes and inhibited expression of some other ones. This manuscript provides the evidence that G. theta rhodopsin genes are at least expressed and opens a possibility to study the encoded proteins. I believe this work can be published in PLOS One, but, unfortunately, the manuscript is very poorly written and needs to be substantially revised to make it comprehensible.

[Reply]

Thank you for your comments. To improve the manuscript written in English, we made use of a professional editing service (Editage: www.editage.com) before resubmission. We reply to your comments as follows.

1) Major issues:

[Comment 1]
Line 182: “To investigate whether a change in pigment composition in culture occurred”

I don’t understand why the Authors measured chlorophyll a content. The fact that photosynthetic pigments are degraded in aging algal cultures is very well known (e.g. [PMID: 27057086]). All cryptophytes contain phicobilins in addition to chlorophyll; in G. theta it is phycoerythrin. As the Authors correctly note, the change of red-brown to green color that they observed in aging G. theta cultures was most likely owing to predominant degradation of phycoerythrin as compared to that of chlorophyll. To test this hypothesis, they would need to measure the contents of both, phycoerythrin and chlorophyll (as it has been done in Ref. [31] in Rhodomonas) rater than chlorophyll alone. But, as Guillardia and Rhodomonas are related organisms, it is very likely that the processes of pigment degradation in them are similar. So, the Authors could simply have hypothesized that the color change they observed under nitrogen-deficient conditions in Guillardia could be explained by preferential degradation of phycoerythrin compared with chlorophyll, as measured in Rhodomonas. In any case, measurements of chlorophyll alone do not seem to produce any relevant information.

[Reply 1]

As you mentioned, nitrogen depletion in algae and cyanobacteria causes degradation of phycobilin and phycoerythrin in the light-harvesting antennae, and quantitative evaluations of these decreases are essentially needed to clarify the changes in pigment composition. However, it was difficult to measure them quantitatively, so we did not measure them in this study. On the other hand, the quantification of chlorophyll a was intended to be an indicator of nitrogen deficiency, which is necessary for future experiments. For this reason, the section on pigment quantification in "Results" was revised as follows.

Line 208: "To investigate the effect of N nutritional status on the pigment content, ..."

In addition, we added to the "discussion" on the need to quantify phycobilin and phycoerythrin to elucidate the changes in pigment composition associated with nitrogen depletion, as follows.

Lines 261 – 262; “Quantitative analysis of phycobilin and phycoerythrin is a topic of future research..”

[Comment 2]
Supplementary Figure 1:

The sequences of Gt150796, Gt107989 and Gt135937 show blanks in the residue positions in the right part of the table. Does it mean that translation of the corresponding transcripts terminated before that? In any case, as determination of the actual transcript sequences is important, the Authors should show an alignment of the entire rhodopsin domains of the encoded proteins in the supplement and deposit their RNA data to a publicly available database. Also, in the text the Authors should comment on comparison of their 29 actual transcripts with the model transcripts predicted by the G. theta genome portal. As the expression constructs used in the previous studies (Refs. [27] and [28]) were based on the latter, possible errors in intron prediction might have influenced some of the results reported therein.

[Reply]

Amino acid sequences used for phylogenetic analysis were from the database, except for Gt_164280 and Gt_120390, which are described below. To clarify this point, we revised the phylogenetic analysis section of the method as follows.

Line 385-386; "The amino acid sequences used for phylogenetic analysis were those registered in NCBI Reference Sequences (RefSeq) except for Gt_120390 and Gt_164280."

Alignments in Fig.1 and S1 Fig. were made by deleting gaps and long loops where the sequence similarity was ambiguous, as described in Methods. Phylogenetic analysis results were uploaded in fasta files rather than in Figure as S1 dataset.

Gt_150796, Gt_107989 and Gt_135937 were truncated on the C-terminal side based on alignment results, with TM7 lost in Gt_150796 and Gt_135937 and TM6 and 7 lost in Gt_107989. As you mentioned whether this sequence is correct is an important question that needs to be examined. However, we could not detect any expression of Gt_150796 and Gt_107989 under our experimental conditions, and we could not detect any expression of Gt_135937 in the RT-PCR experiments shown in Fig. 4. Therefore, we did not determine the mRNA sequences for these genes.

Among the expressed genes, the sequences of Gt_164280 (GtCCR4) and Gt_120390 were deciphered and found to be different from the sequences in the database. We decoded the partial sequences of the mRNAs and registered in a public database (Accession numbers are MF039475 and LC591948, respectively). We mentioned the comparison of these sequences with model transcripts and genomic sequences in the results as follows. Sequence comparisons of the transcripts and the predicted amino acid sequences were described in S2 Fig. and S3 Fig. The original S2 Fig. was renumbered to S4 Fig.

Lines 132-150: “Using cDNAs derived from the extracted mRNAs, we re-analyzed the amino acid coding regions of Gt_164280 and Gt_120390. Gt_164280 was compared to the model transcript in the RefSeq database (S2 Fig.). The nucleotides corresponding to the 451st to 462nd base were deleted in the model transcript and the 710th, 816th and 869th bases of re-analyzed sequence were replaced from T to C, T to C and G to A, respectively (S2a Fig.). As a result, a four-amino acid insertion occurred in the amino acid sequence corresponding to TM4 in the predicted protein from the re-sequenced gene compared to that of the model transcript (S2b Fig.). In re-analyzed sequence, Met234 and Ser287 of the model transcript were replaced to Thr and Gly, respectively. Gt_120390 was compared to both the model transcript and the genomic sequence (S3 Fig.). The seventh exon predicted from the genomic sequence was an exact match to the mRNA sequence we determined, but the model transcript had a large deletion of 144 bases (S3a Fig.). On the other hand, the mRNA we determined had a 44-base extension after the 10th exon to the intron region and then a stop codon was appeared. The 11th exon was deleted in our sequence. As a result, the transmembrane region of the predicted protein from the re-analyzed gene was consistent with that of the model transcript, but a 48-residue insertion and 14-residue amino acid substitution occurred in the C-terminal extension (S3b Fig.). The C-terminal six residues were truncated in the predicted protein from re-analyzed transcript. “.

2) Lesser issues:

[Comment 1]
Lines 49-50: “In 2002, two rhodopsins in green algae Chlamydomonas reinhardtii were found to act as light-gated ion channels [4].”

Ref. [4] reports channel properties of only one C. reinhardtii rhodopsin, ChR1. Also, please change "algae" to "the alga".

[Reply 1]

We revised the manuscript as you pointed.

[Comment 2]
Line 55-56: “Moreover, a new group of rhodopsin named heliorhodopsin also found in nature [8].”

Nowadays “new groups of rhodopsins” are found in nature almost every month. I believe the Authors should briefly explain here what is so new about heliorhodopsins that merits mentioning them in the introduction.

[Reply 2]

As you mentioned, we have revised the manuscript on the novelty of HeR as follows.

Lines 58-60: “Heliorhodopsins display less than 15% sequence identity with microbial and animal rhodopsins. In the membrane, HeR is oriented in the opposite direction to the other rhodopsins.”

Although we did not target the expression analysis in this paper, there are two heliorhodopsin-like genes in *G.theta*. Therefore, the heliorhodopsin-like genes in *G.theta* were mentioned in "Results" as follows.

Lines 151-153; “*Guillardia theta* also carries two heliorhodopsin-like genes (XM_005821825 and XM_005823076); however, we did not analyze the expression of these heliorhodopsins in this study*.*”

[Comment 3]
Lines 57-58: “…microbial rhodopsins are widely distributed in not only bacteria but also cyanobacteria, algae, protozoa, and giant viruses [9].”

Ref. [9] reviews only prokaryotic rhodopsins, whereas algae and protozoa are eukaryotes. Moreover, to the best of my knowledge, there are no published reports of rhodopsins in protozoa. The attribution of a rhodopsin transcript to the marine ciliate Tiarina fusus [PMID: 31320556] was erroneous; in fact, this rhodopsin was derived from a cryptophyte alga used to feed Tiarina in culture.

[Reply 3]

As you mentioned, Pinhassi J, et al (2016) reviews only prokaryotic rhodopsins. We added references of Philosof et al, (2013; reference number is 10) and Govorunova et al., (2017; reference number is 11). We couldn't find a suitable paper that mentioned Protozoa rhodopsin, so we followed your point and removed "protozoa" from the manuscript.

[Comment 4]
Line 64: “photosensor for phototaxis in H. salinarum”

Phototaxis is the ability to track the direction of light, of which H. salinarum is not capable. Please change “phototaxis” to “photomotility”. Please also change the past tense to the present tense in the entire paragraph, i.e. “Sensory rhodopsins (SR) and channel rhodopsins act as …”, not “acted”.

[Reply 4]

We revised the manuscript as you pointed.

[Comment 5]
Line 74: “flabobacteria Doktonia sp. MED134”

This genus name is spelled Dokdonia.

[Reply 5]

We revised the manuscript as you pointed.

[Comment 6]
Line 76-77: “These results suggest that microbial rhodopsins are related to primary metabolisms”

This conclusion can only be drawn about rhodopsins in Dokdonia, not about the entire superfamily. Furthermore, this paragraph needs to be rewritten for clarity. Start with “Nitrogen is important nutrition as a source of metabolites including amino acids” and then describe the results in Dokdonia. Delete the sentence “Algae contribute suppressing an increase in CO2 concentration in the ocean by fixing CO2 by photosynthesis” as trivial and not relevant for this paragraph.

[Reply 6]

We revised the manuscript as follows.

Lines 79-80: “Nitrogen is an important source of metabolites including amino acids. Gene expression analysis of…”

Lines 83-85: “… These results suggest that microbial rhodopsins are related to primary metabolisms processes in *Dokdonia* sp. MED134, … “

We deleted the sentence “Algae contribute suppressing an increase in CO_2_ concentration in the ocean by fixing CO_2_ by photosynthesis.”

[Comment 7]
Lines 252-253: “which have been reported in the past as sensory rhodopsin related to the phototaxis”

Please change “reported” to “suggested”. The Authors apparently mean Ref. [26], in which it has been shown that the absorption maximum of heterologously expressed and purified GtR1 lies within the broad spectral region to which G. theta motility is sensitive. This result at best justifies only a suggestion that GtR1 might contribute to phototaxis, but by no means can be considered as a “report” of its function as the receptor for phototaxis! Such conclusion would require e.g. gene knockdown studies. Also, since 2005 both CCRs and ACRs have been discovered in G. theta, and these proteins are much more likely candidates for phototaxis receptors than GtR1.

[Reply 7]

We revised the manuscript as you pointed.

3) Some of the style and grammar problems (but there are many more throughout the manuscript – please take care of them as well):

[Comment 1]
Lines 40-42: “Microbial rhodopsins are the light-receiving membrane proteins, which act as a light-driven ion pump, a light-driven ion channel, light-driven enzyme, and photosensor [1].”

Please consistently use either plural or singular in this sentence and throughout the entire paragraph.

[Reply 1]

We revised the manuscript as you pointed.

[Comment 2]
Line 58: “On the other hand, there are about 600 genes”

The expression “on the other hand” is unsuitable here; please change to “in addition” and add “known” after “there are”.

[Reply 2]

In consideration of this comment as well as your other comments (comment number 3 and 15), we deleted this sentence

[Comment 3]
Lines 59-60: “As physiological roles, they are related in energy production…”

This sentence follows immediately after the sentence about Rh-noK, so the reader assumes that it describes their functions! To avoid this confusion, please move the sentence about Rh-noK to the end of this paragraph.

[Reply 3]

We moved the sentences about Rh-noK to lines 70-74 and revised as follows.
“Recent advances in genome research have led to the discovery of many proteins that are similar to microbial rhodopsin but lack the conserved retinal-binding lysine residue (Rh-noK). There are currently 5,558 known genes of microbial rhodopsins, including HeRs, of which approximately 600 are Rh-noK. [19]. Some Rh-noK genes were tandemly arranged with PR and retinal biosynthesis genes forming a putative operon [19].”

[Comment 4]
Line 68: “…there were many kinds of research on the molecular properties of microbial rhodopsin …”

Please change to “…molecular properties of microbial rhodopsins are studied by many approaches…”

[Reply 4]

We revised the manuscript as you pointed.

[Comment 5]
Line 86-87: “Cryptophytes are unicellular algae living in ubiquitously from marine to freshwater environments”

Change to “Cryptophytes are unicellular algae ubiquitously found in marine and freshwater habitats”.

[Reply 5]

We revised the manuscript as you pointed.

[Comment 6]

Lines 88-89: “…a secondary plastid that had been laterally transferred from…”

The term “lateral transfer” refers to genes, not entire organelles. Please change to “acquired”.

[Reply 6]

We revised the manuscript as you pointed.

[Comment 7]
Line 90-91: “The reason for the importance of the evolution of plastid…”

Please change to “Owing to the importance…”.

[Reply 7]

We revised the manuscript as you pointed.

[Comment 8]
Lines 92-93: “The nuclear genome of G. theta codes many genes similar to microbial rhodopsins in its nuclear genome”.

Please delete “in its nuclear genome” at the end of this sentence.

[Reply 8]

We revised the manuscript as you pointed.

[Comment 9]
Lines 94-95: “many of them were revealed neither their molecular functions nor physiological functions”

Please change to “Molecular properties and physiological functions of many G. theta rhodopsins are currently not known”.

[Reply 9]

We revised the manuscript as you pointed.

[Comment 10]
Line 109: Please change “phylogenic” to “phylogenetic” here and throughout the manuscript.

[Reply 10]

We revised the manuscript as you pointed.

[Comment 11]
Line 131: “The name of representative rhodopsins indicated as follows”.

Please change to “Abbreviations:”or delete this sentence altogether.

[Reply 11]

We deleted the sentence as you pointed.

[Comment 12]
Line 211: “While there were 15 genes decreased the expression level in N depletion”

Please change to “The expression level of 15 genes decreased upon N depletion”.

[Reply 12]

We revised the manuscript as you pointed.

[Comment 14]
Line 254: Please substitute the word “ref” with the corresponding reference number.

[Reply 14]

We cited a manuscript (Sineshchekov et al, 2009; reference number is 28) as follows.“…in the past as sensory rhodopsin related to the phototaxis [28], but…”

[Comment 15]
Lines 278-283: Please move these sentences to Introduction.

[Reply 15]

We moved these sentences about Rh-noK to Introduction and revised them as described in the comment 3.

[Comment 16]
Lines 284-285: Please delete the words “In a similar case”. There is nothing similar between the rest of the sentence and the preceding text. The similar case is that some GAF domains have no critical cysteine, as some rhodopsin-like proteins have no critical lysine; please change the text accordingly.

[Reply 16]

Based on your comments, we revised the manuscript as follows.

Lines 309-317; “…Rh-noK does not have a lysine residue that binds the chromophore, all-trans retinal, so it is likely to function without binding the all-trans retinal. Photoreceptor proteins such as phytochrome and cyanobacteriochrome have GAF domains that bind chromophore phytochromobilin [35]. The chromophore binds to conserved cysteine residues in the GAF domain. However, as Rh-noK has no conserved lysine residues, there are also proteins that have a GAF domain but no conserved cysteine residues [36]. Although these proteins could not bind any chromophore, they have many diverse functions, such as a sensor for sodium-ion [37] and chloride ion concentrations [36]. Many Rh-noK genes have been found …”.
