# Supplementary material for: Expression analysis of microbial rhodopsin-like genes in Guillardia theta
Source: PLoS One. 2020 Dec 3;15(12):e0243387. doi: 10.1371/journal.pone.0243387 (PMC7714340; doi:10.1371/journal.pone.0243387)
Supplement: Attachment — Submitted filename: Konno_et_al_Response to reviewer#2.docx [file pone.0243387.s007.docx]

**Response to Reviewer 2:**

Thank you for your crucial reading of our manuscript again and nice comments on it. We will reply to your comments as follows.

[Comments]
 The Authors have much improved the manuscript during revision. They have answered all my questions and also corrected most of stylistic problems. I recommend the revised version for publication in PLOS One, although I still have a few comments, as indicated below. I am sure the Authors will agree to make these suggested changes, so I do not think there is a need to send the manuscript to me again.

[Reply]

Thank you for your comments. We completely agreed the reviewer’s comments, so we reply to your comments as follows.

[Comment 1]
Lines 50-52: “In 2002, two rhodopsins in the green alga Chlamydomonas reinharditii were found to act as light-gated ion channels [4]”.

As I have already indicated in my review of the original version, Ref. [4] reports only ONE channelrhodopsin from C. reinhardtii. The second one from this organism was described in a different paper in 2003 (PMID: 14615590). The Authors state in their response letter that they have corrected this error, but in fact they have not. Please change the plural in this sentence to singular (“a rhodopsin” and “was found”), or keep the plural and add the above-mentioned paper to the reference list.

[Reply 1]

Thank you for pointing out our error. As you mentioned, we revised the manuscript as follows.

Line 51-52: “… a rhodopsins in the green alga *Chlamydomonas reinharditii* was found to act as a light-gated ion channel [4]. ”

[Comment 2]

Lines 106-107: “In G. theta, some of the rhodopsin-like genes are related to the regulation of N-assimilation by energy production”.

If this is the conclusion that the Authors draw from their present study (as I understand it is), this sentence should be moved to the next paragraph, e.g. “In this study, we investigated the expression pattern of rhodopsin-like genes in G. theta under various growth conditions. We show that some of the rhodopsin-like genes are related to the regulation of N-assimilation by energy production in this organism.” If this is a conclusion from some earlier studies, a proper reference should be given.

[Reply 2]

Thank you for your useful comment. As you mentioned, we moved to the sentence to next paragraph and revised the manuscript as follows.

Line 110-111: “… We show that some of the rhodopsin-like genes are related to the regulation of N-assimilation by energy production in this organism.”

[Comment 3]

Lines 222-223: “Guillardia theta has a predicting beta-carotene 15, 15'-monooxygenase gene”

Please change “predicting” to “predicted”.

[Reply 3]

We revised the manuscript as you pointed.
